# Relaxin Treatment in an Ang-II-Based Transgenic Preeclamptic-Rat Model

**DOI:** 10.1371/journal.pone.0150743

**Published:** 2016-03-10

**Authors:** Nadine Haase, Michaela Golic, Florian Herse, Julianna Rugor, Dominik Linz, Maria Emilia Solano, Dominik N. Müller, Ralf Dechend

**Affiliations:** 1 Experimental and Clinical Research Center, a joint cooperation between the Max Delbrück Center for Molecular Medicine in the Helmholtz and the Charité Medical Faculty, Berlin, Germany; 2 Klinik für Innere Medizin III, Kardiologie, Angiologie und Internistische Intensivmedizin, Universitätsklinikum des Saarlandes, Homburg/Saar, Germany; 3 Department of Obstetrics and Fetal Medicine, Laboratory for Experimental Feto-Maternal Medicine, University Medical Center Hamburg-Eppendorf, Hamburg, Germany; 4 HELIOS-Klinikum Berlin, Berlin, Germany; University Medical Center Utrecht, NETHERLANDS

## Abstract

Relaxin is a peptide related to pregnancy that induces nitric oxide-related and gelatinase-related effects, allowing vasodilation and pregnancy-related adjustments permitting parturition to occur. Relaxin controls the hemodynamic and renovascular adaptive changes that occur during pregnancy. Interest has evolved regarding relaxin and a therapeutic principle in preeclampsia and heart failure. Preeclampsia is a pregnancy disorder, featuring hypertension, proteinuria and placental anomalies. We investigated relaxin in an established transgenic rat model of preeclampsia, where the phenotype is induced by angiotensin (Ang)-II production in mid pregnancy. We gave recombinant relaxin to preeclamtic rats at day 9 of gestation. Hypertension and proteinuria was not ameliorated after relaxin administration. Intrauterine growth retardation of the fetus was unaltered by relaxin. Heart-rate responses and relaxin levels documented drug effects. In this Ang-II-based model of preeclampsia, we could not show a salubrious effect on preeclampsia.

## Introduction

Preeclampsia is one of the leading causes of maternal and fetal mortality and morbidity, complicating 3–8% of pregnancies.[1] The disease is characterized by new onset of maternal hypertension after 20th week of gestation and proteinuria or in association with thrombocytopenia, impaired liver function, the new development of renal insufficiency, pulmonary edema, or new-onset of cerebral or visual disturbances.[2] Preeclampsia originates in the placenta, but the underlying etiology is complex and probably heterogeneous in origin.[1]

Although the molecular mechanisms leading to preeclampsia remain largely unknown, the disturbed placental function in early pregnancy is a major contributor in the leading hypotheses. Impaired endovascular trophoblast invasion and vascular remodeling of the spiral artery is an early, but not necessarily the primary, pathology involved in preeclampsia.[3] The clinical syndrome “preeclampsia” might result from imbalance between factors produced by the placenta in response to abnormal placentation and maternal adaptation to them leading to endothelial dysfunction and increased vascular reactivity.[4] At this stage profound systemic vasoconstriction is a prominent feature of preeclampsia with hypertension, renal dysfunction, and multiple organ failure. Reduced blood flow to the placenta inducing soluble, vasoactive factors, such as sFlt1 might be the underlying mechanism.[5]

The various functions of relaxin suggest that administration of this protein to women with preeclampsia may be an interesting therapeutic approach.[6] Relaxin is a protein hormone of about 6000 Da first described in 1926. [7] Relaxin regulates maternal adaptations to pregnancy with several effects potentially relevant to the treatment of acute heart failure, including increased arterial compliance, cardiac output, and renal blood flow.[8] The effects of relaxin include the production of nitric oxide, inhibition of the renin-angiotensin and endothelin system, production of VEGF, and induction of gelatinases, such as matrix-metalloproteinases. These effects lead to systemic and renal vasodilation, increased arterial compliance, and other vascular changes which have been reviewed in detail.[9] The molecular mechanisms of relaxin vasodilation depend on the duration of hormone exposure. There are rapid and sustained vasodilatory responses.[10] Conrad et al indicated that the vasodilatory responses of relaxin are transduced by a specific G protein-coupled receptor, RXFP and are mediated by Gαi/o protein coupling to phosphatidylinositol 3-kinase/Akt (protein kinase B)–dependent phosphorylation and activation of endothelial nitric oxide synthase (NOS).[10]

We tested the effects of relaxin in a transgenic rat model of preeclampsia, which is generated by mating female rats transgenic for human angiotensinogen with rats transgenic for human renin. [11, 12] Dams exhibit an increase in blood pressure from 100/80 mm Hg to 180/140 mm Hg and develop proteinuria at the end of pregnancy. The model is associated with altered placentation, modified resistance index, and endothelial dysfunction. Although upregulation of the renin-angiontensin system is the underlaying mechanism in this model, a disturbed prostacyclin:thromboxane ratio is an important mediator of the increased vasoconstriction and abnormal vasocreactivity.[13] The uteroplacental units present a pathological endovascular and interstitial trophoblast invasion and display an altered vascular remodeling.[14–16].

## Methods

Female Sprague-Dawley (SD) rats harboring the human angiotensinogen gene [TGR(hAogen)L1623] were mated with male SD rats bearing the human renin gene [TGR(hRen)L10J], after the implantation of radiotelemetry pressure transducers (TA11PA-C20, Data Sciences International, La Jolla, Calif) as described before.[17, 18]. The radiotelemetry pressure transducers were implanted in the abdominal cavity of the rat under isoflurane anesthesia (isoflurane dose of 1.6% in 400 ml/min air flow), with the transducer connected capillary tubing anchored in the lumen of the abdominal aorta. Before the implantation the zero offset was measured and the unit was soaked in 0.9% NaCl. Before surgery, the animal receives an analgesic dose of carprofen (Rimadyl®, 5mg/kg, subcutane). Xylocaine 2% Jelly was used as local surface anesthesia after surgery. Animals were allowed to recover for 10 days. We were aware that female hAogen rats mated with male hRen rats would develop the preeclamptic syndrome.[17, 18] The observation of plug in the vagina is indicating the day 1 of pregnancy. On gestational day 9, hAogen transgenic dams were randomly assigned to 2 experimental groups: vehicle (20 mM sodium acetate, pH 5.0, n = 5) or relaxin (n = 5), at a dose of 2 μg/h of relaxin by subcutaneous osmotic minipump (Alzet, Typ 2002) until day 21 of gestation. Subcutaneous implantation of osmotic pumps was done under isoflurane anesthesia (isoflurane dose of 1.9% in 400 ml/min air flow). Twenty-four-hour urine samples were collected in metabolic cages at day 17/18 of gestation. Rats were killed at day 21 of gestation by decapitation after prior anesthesia with isoflurane. The fetuses and organs were removed and weighed. Serum and plasma samples were collected. Sprague-Dawley females were mated with Sprague-Dawley males as controls, namely pregnant Sprague-Dawley (SD). SD rats were killed at day 21 of gestation. Serum and plasma samples, as well as uteroplacental units were collected. Local authorities approved the studies (State Office of Health and Social Affairs Berlin; permit number: G0015/13) and all procedures were done according to guidelines from the American Physiological Society. All surgery was performed under isoflurane anesthesia, and all efforts were made to minimize suffering. Human recombinant relaxin was kindly provided by Novartis, Switzerland and has been shown previously to be bioactive in rodents.[19]

Urinary rat albumin was measured with a commercially available ELISA (CellTrend, Germany). Serum concentration of human (Immundiagnostik, Germany) and rat relaxin (MyBioSource, USA) was also measured with a commercially available ELISA. Serum cystatin C was measured with a commercially available ELISA (BioVendor, Germany) and serum creatinine were determined by an automated clinical method.

Analysis of renal gene expression: Total mRNA was isolated with TRIZOL followed by the Qiagen protocol, and TaqMan reverse-transcription polymerase chain reaction (RT-PCR) was performed as recommended by the manufacturer. Quantitative analysis of target mRNA expression was performed with real-time PCR using the relative standard curve method. Real-time PCR was detected on ABI 7500 Fast Sequence Detection System (Applied Biosystems) and analyzed by 7500 Fast System Software (Applied Biosystems). Primers and probes were designed with PrimerExpress 3.0 (Applied Biosystems) and synthesized by Biotez (Germany). The expression levels of the target gene NGAL in the kidney was normalized to 18S as the endogenous control. The following primers were used for reverse transcriptase, PCR amplification and detection: NGAL: CAGGGCAGGTGGTTCGTT (forward), AGCGGCTTTGTCTTTCTTTCTG (reverse), CGGCCTGGCAGCGAATGC (probe). 18S: ACATCCAAGGAAGGCAGCAG (forward), TTTTCGTCACTACCTCCCCG (reverse), CGCGCAAATTACCCACT CCCGAC (probe).

Angiotensin metabolites levels in the serum were measured by Attoquant Diagnostics GmbH (Vienna, Austria). Using mass spectrometry they investigated Ang II levels and the following Ang metabolites:, Ang 1–10 (Ang I), Ang 1–8 (Ang II), Ang 1–7 (MAS receptor agonist), Ang 2–8 (Ang III), and Ang 3–8 (Ang IV) as well as Ang 1–5, Ang 1–9, Ang 2–7, Ang 2–10 and Ang 3–7. Samples were spiked with 100 pg/ml stable-isotope-labeled internal standards and subjected to solid-phase extraction using Sep-Pak cartridges (Waters) according to manufacturer’s protocol. Following elution and solvent evaporation, samples were reconstituted in 50 μl 50% acetonitrile/0.1% formic acid and subjected to LC-MS/MS analysis using a reversed-phase analytical column (Luna C18, Phenomenex) using a gradient ranging from 10% acetonitrile/0.1% formic acid to 70% acetonitrile/0.1% formic acid in 9 minutes. The eluate was analyzed in line with a QTRAP-4000 mass spectrometer (AB Sciex) operated in the MRM mode using dwell times of 25 msec at a cone voltage of 4000 volts and a source temperature of 300°C. For each peptide and corresponding internal standards, two different mass transitions were measured. Ang II peptide concentrations were calculated by relating endogenous peptide signals to internal standard signals.

Rat uteroplacental units were fixed in buffer according to Beckstead J.H.[20] truncated from two lateral placental parts and embedded in paraffin. Paraffin embedded tissue was cut into 3 μm histological sections at the mid sagittal plane. Tissue sections were deparaffinized, rinsed in distilled water, and dehydrated twice in ethanol 70%. Masson Trichrome staining were performed using standard protocols. Image acquisition was performed using a slide scanner (Mirax Midi Slide Scanner) For the interpretation of vascular remodeling of spiral arteries the outer diameter (OD) and luminal diameter (LD) of each vessel were measured at the point of the largest OD using Pannoramic Viewer (3D Histech). The ratio of LD:OD was used to compare the proportionate vessel wall thickness of the spiral arteries in the mesometrial triangle.

All data are presented as means ± SEM. Group differences were analyzed by t test, Mann–Whitney U test, or ANOVA with Bonferroni post hoc test, as appropriate. A value of p < 0.05 was considered statistically significant.

## Results

**Fig 1:**
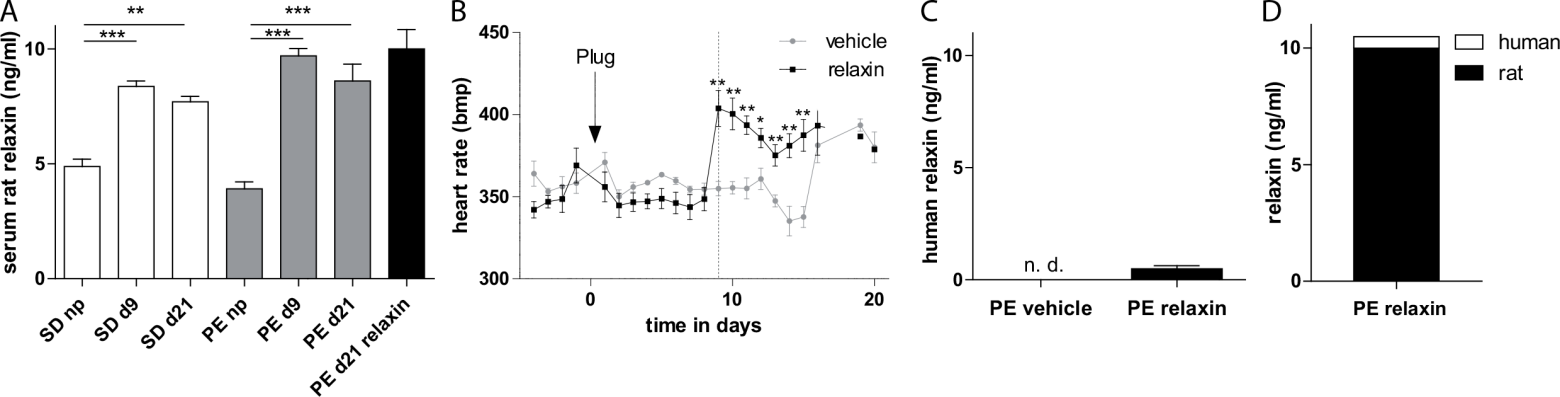
Effect of relaxin on heart rate and relaxin serum concentration. (A) Rat relaxin was increased during pregnancy in transgenic and non-transgenic control rats. (B) Telemetrically measured heart rates. Relaxin treatment leads to an immediately increase in heart rate compared to vehicle treated rats. The arrow with plug indicates the observation of plug in the vagina, indicating day 1 of pregnancy. Start of treatment is indicated by the dotted line. (C) Human recombinant relaxin was present in the relaxin and not detectable (n.d.) in vehicle treated animals. All results are expressed as mean ± SEM of 5 animals per group, * p<0.05. (D) Illustration of absolute relaxin amount by the addition of endogenous and exogenous relaxin levels (demonstrated in A and C).

In pregnant rats naturally occurring rat relaxin serum levels were increased during pregnancy nevertheless no differences in relaxin serum concentration between nontransgenic Sprague-Dawley (SD) rats and transgenic preeclamptic rats were observed (Fig 1A). Telemetric measured heart rate values of vehicle and relaxin preeclamptic rats are shown in Fig 1B. Relaxin treated preeclamptic rats developed significantly increased heart rates directly after the administration at gestational day 9 (355.0 ± 4.4 bmp vehicle vs. 403.8 ± 11.0 bpm relaxin, p = 0.0033) and had sustained until day 15. Treatment with recombinant human relaxin led to serum concentrations that were 500 pg/ml or so at gestational day 21. We detected no human relaxin in vehicle-treated preeclamptic rats (Fig 1C). The continuously infused human relaxin by osmotic minipump at 2 μg/h for 12 days led only to a slight elevation of 4.9% of the endogenous relaxin amount (Fig 1D).

**Fig 2:**
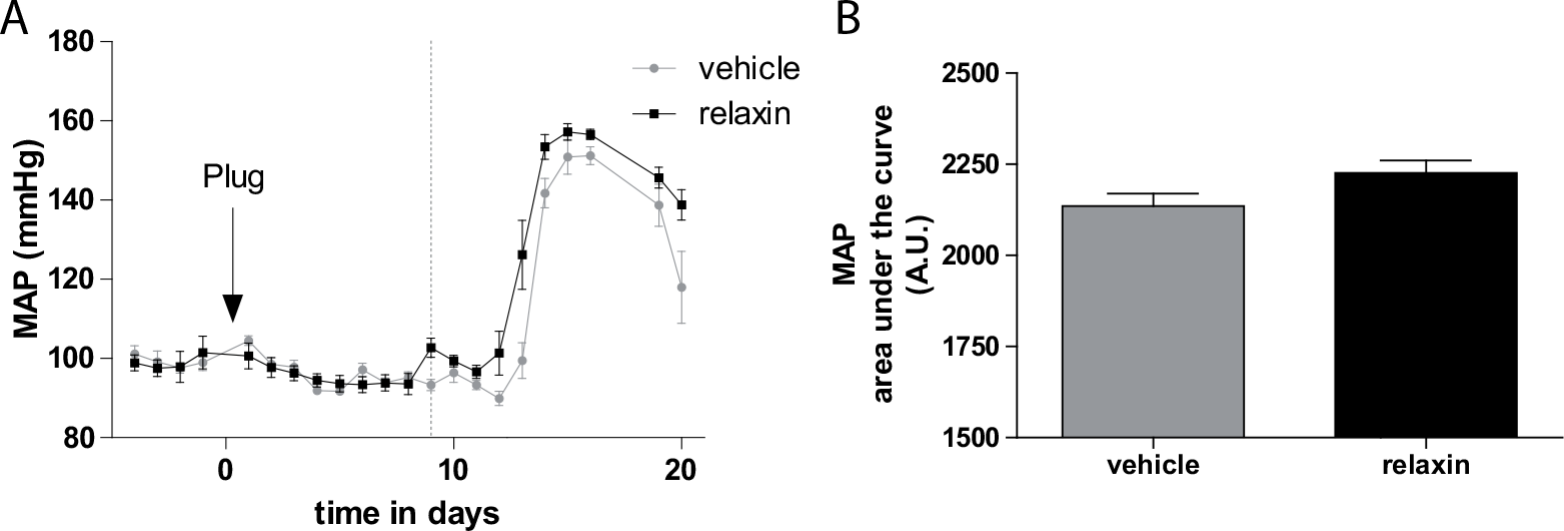
Effect of relaxin on blood pressure. (A) Telemetrically measured mean arterial blood pressures. Hypertension occurred abruptly at day 13 of gestation and at the end of pregnancy there was some decline in vehicle treated preeclamtic rat model. Relaxin treatment did not prevent the rise in blood pressure. The arrow with plug indicates the observation of plug in the vagina, indicating day 1 of pregnancy. Start of treatment is indicated by the dotted line. (B) Calculated mean arterial blood pressure area under the curve showed no differences. Results are expressed as mean ± SEM of 5 animals per group.

Telemetric measured mean arterial blood pressure (MAP) values of vehicle and relaxin treated hAogen transgenic dams mated with a male hRen transgenic rats are shown in Fig 2A. Vehicle treated preeclamptic rats developed hypertension abruptly at gestational day 13 and had sustained hypertension until shortly before delivery, when blood pressure decreased slightly. Relaxin treatment begun even before hypertension developed on gestational day 9 and did not prevent the increase in blood pressure in the last third of pregnancy (MAP on day16 of gestation: 151.1 ± 2.2 mmHg vehicle vs. 156.6 ± 1.3 mmHg relaxin). Mean arterial blood pressure area under the curve was calculated and yielded no differences between vehicle and relaxin treated rats (Fig 2B).

**Fig 3:**
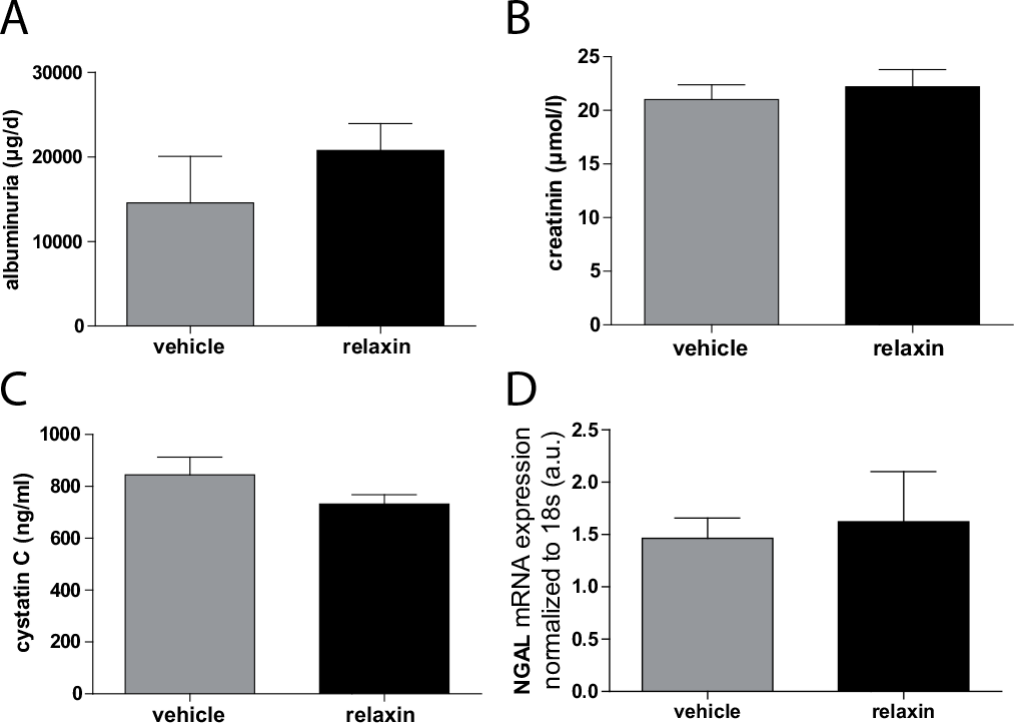
**Effect of relaxin on renal function** (A) Albuminuria was present in vehicle treated animals and was not changed by realxin. (B) Serum creatinine and (C) cystatin C were not altered after administration of relaxin. (D) The expression of renal NGAL was not different between vehicle and relaxin treated rats. All results are expressed as mean ± SEM of 5 animals per group.

**Fig 4:**
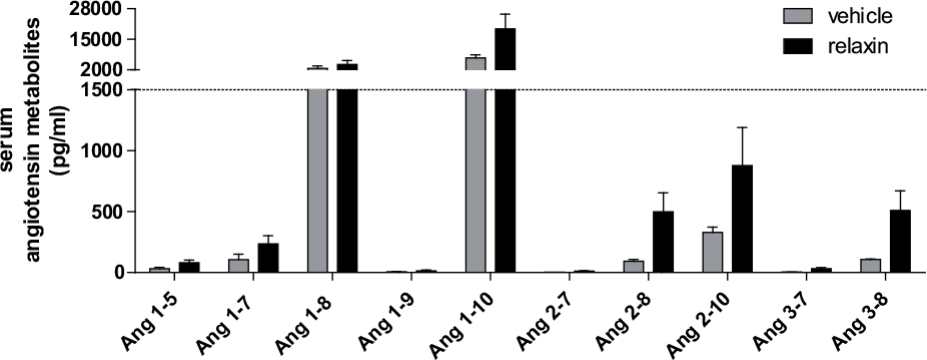
Effect of relaxin on angiotensin metabolites. Serum angiotensin metabolites levels were not significant different between vehicle and relaxin treated rats. All results are expressed as mean ± SEM (n = 3 vehicle, n = 3 relaxin).

Albuminuria (Fig 3A) that occurred in vehicle treated preeclamptic rats in the third trimester was not ameliorated by relaxin (14.6 ± 5.5 mg/d vehicle vs. 20.8 ± 3.8 mg/d relaxin). Furthermore, relaxin administration in preeclamtic rats had no influence on serum creatinine (Fig 3B, 21.0 ± 1.4 μmol/l vehicle vs. 22.2 ± 1.6 μmol/l relaxin) and cystatin C (Fig 3C, 844.8 ± 68.5 ng/ml vehicle vs. 732.2 ± 36,0 ng/ml relaxin) concentration compared to vehicle group. Moreover there was no influence on renal NGAL mRNA expression by the administration of relaxin (Fig 3D). To explore the influence of relaxin on renin-angiotensin system (RAS) we measured serum angiotensin metabolites mass spectroscopy. We could not detect significantly changes in the amount of all of them in the circulation between vehicle and relaxin treated rats (Fig 4).

**Fig 5:**
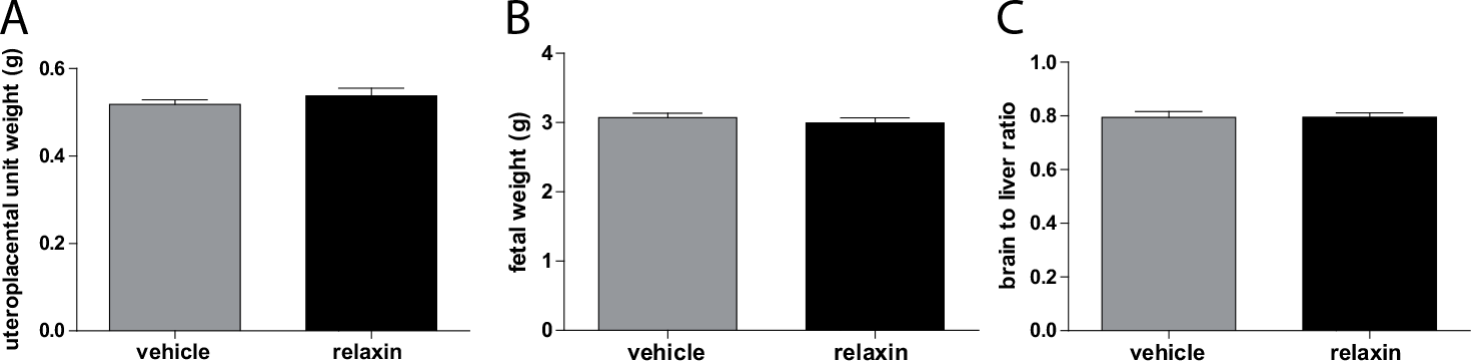
Effect of relaxin on fetal phenotype. Fetal weight (A) Uteroplacental unit weight (B), and brain to liver weight ratio (C) in vehicle and relaxin treated hAogen TGR dams at gestational day 21. Fetal (A) and uteroplacental unit (B) weights, as well as brain to liver ratios (C) were similar between vehicle and relaxin treated preeclamptic rats. All results are expressed as mean ± SEM (n = 51 vehicle, n = 47 relaxin).

**Fig 6:**
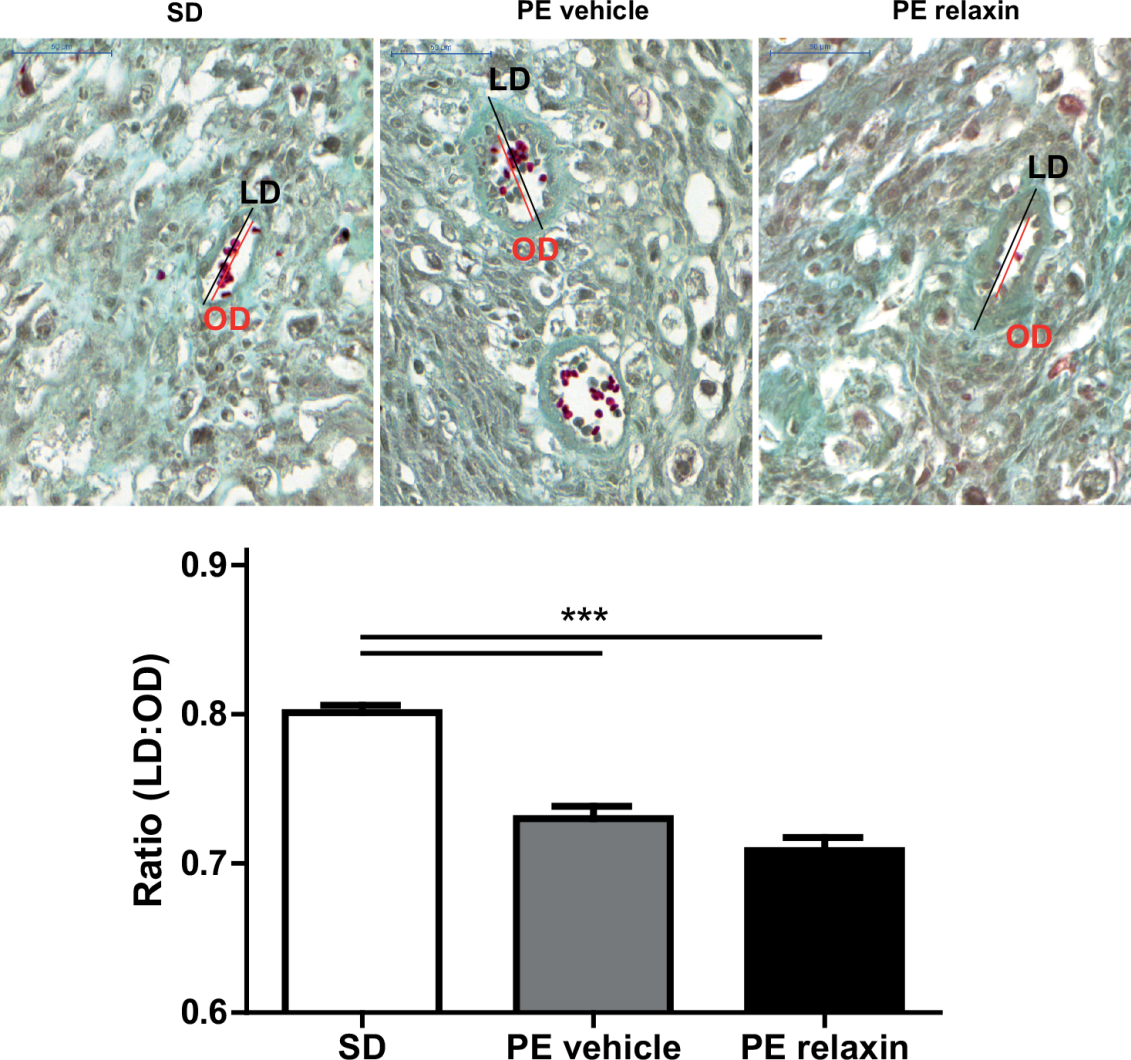
Effect of relaxin on spiral artery remodeling. Representative images of Masson Trichome-stained uteroplacental unit sections of SD, vehicle and relaxin treated PE rats at gestational day 21. Maternal vessels in the mesometrial triangle of SD rats (upper left panel) showed thin arterial walls. In contrast, vessels in this region of vehicle (upper middle panel) and relaxin (upper right panel) treated PE rats a thickened arterial wall. These differences are summarized as ratios of the inner lumen to-outer diameter ratios (LD/OD) (lower panel). The outer diameter (OD, red line) and luminal diameter (LD, black line) of each vessel were measured at the point of the largest OD All results are expressed as mean ± SEM (***p<0.001, SD vs. PE vehicle, SD vs. PE relaxin; Kruskal-Wallis test with Dunn’s post hoc; n = 354 SD, n = 246 PE vehicle, n = 281 PE relaxin).

The number of live fetuses was similar between the groups (11.2 ± 1.3 vehicle vs. 9.4 ± 1.9 relaxin). Although relaxin treatment seemed to have a partial deleterious effect on the outcome of these pregnancies: the percentage of resorptions per pregnant female rat was higher. Fetal and uteroplacental unit weights are shown in Fig 5. Fetal weights (Fig 5A) were unaffected by relaxin treatment (3.072 ± 0.062 g vehicle vs. 2.996 ± 0.072 g relaxin). Uteroplacental unit weights (Fig 5B) were also unchanged in vehicle versus relaxin treated preeclamptic rats (0.518 ± 0.011 g vehicle vs. 0.537 ± 0.017 g relaxin). To characterize intrauterine growth retardation (IUGR), we investigated the brain to liver ratio in the fetuses (Fig 5C). Relaxin treatment had no influence on the brain to liver ratio consistent with IUGR (0.795 ± 0.021 vehicle vs. 0.795 ± 0.016 relaxin). Moreover spiral artery remodeling of the uteroplacental unit of preeclamptic rats was not affected by the administration of relaxin. Trichrome staining of uteroplacental units revealed that SD mesometrial triangles had thin-walled spiral arteries compared to vehicle and relaxin treated PE rats. The vessels in this region of vehicle and relaxin treated rats showed a significant thickened arterial wall (Fig 6).

## Discussion

Relaxin did not ameliorate the preeclamptic phenotype in our transgenic rat model although it showed biological activity. Relaxin treatment had no influence on blood pressure, urinary albumin excretion. Furthermore, no improvement of intra-uterine growth retardation and vascular remodeling was observed.

Our rodent model of preeclampsia is based on the secretion of active renin from the placenta or the fetus, which interacts with circulating maternal *h*AGT leading to increased circulating and local angiotensin II. In normal pregnancy relaxin is one of the leading hormones leading to remarkable maternal cardiovascular adaptation and attenuation to systemic pressor response to vasoconstrictors, including angiotensin II and norepinephrine [21]. The rationale to propose relaxin administration to women with preeclampsia is based on the hormone’s vasodilatory attributes, which subsequently might improve organ perfusion. Different pathways modulated by relaxin lead to a potent augmentation of systemic and renal vasodilation by reducing the myogenic reactivity in small renal arteries [22]. Relaxin can directly act on both arterial smooth muscle cells as well as on endothelial cells. Increased production of metalloproteinases, and the generation of endothelin, which then acts via endothelin receptors to activate cGMP and NO are important mechanisms [23]. Moreover, there is a rapid relaxin response in these vessels involving RXFP1 coupling to PI3-kinase and the phosphorylation/activation of eNOS [19]. Nevertheless relaxin did not reduce the preeclamptic phenotype, leaving the authors puzzled by the negative results.

However, the Ang II mediated vasoconstriction might be too striking for relaxin to show it´s positive vasodilating properties and the relaxin dosages archived cannot counteract Ang II action. The described mechanisms should counteract the detrimental effects of Ang II on the renal circulatory response to Ang II [24]. But if hypertension is not reduced, the protective effects of relaxin could also be harmful, leading to progressive loss of autoregulation and increased pressure injury. In normal pregnant rats without hypertension, Smith et al demonstrated that renal autoregulation remained intact in pregnant rat although relaxin inhibited myogenic constriction of renal interlobar arteries [25]. This pathomechanism might be different if Ang II is chronically up regulated in pregnant rats as in our model.

Relaxin is able to stimulate several immune cell types, particularly in the endometrium [26]. Piccinni et al showed that relaxin activates resident T cells into Th1-like effector cells producing IFNγ [27]. The potent modulation of a pro-inflammatory cytokine network by relaxin might be important for appropriate implantation and placentation [28]. However, the importance of these mechanisms in a pro-inflammatory surrounding during preeclampsia is not known and might also be implicated into the negative result we observed.

In rats, circulating relaxin is detectable on gestational days 8 or 9 [29]. Our transgenic preeclamptic rats showed similar circulating relaxin during pregnancy like nontrangenic rats. In human there are also no differences in serum relaxin levels between women with preeclampsia and uncomplicated pregnancy [30, 31]. However the mode of action of relaxin suggests beneficial effects in preeclampsia [32], but not many studies have actually investigated relaxin in preeclampsia. No human trial of relaxin has been performed in human preeclampsia. Unemori et al have presented a rationale and design of a Phase I Safety Study, which has been launched in the USA [6]. Lafayette et al. observed no significant correlation between serum relaxin concentration and renal function in preeclamptic women in the early postpartum period [30]. The authors conclude that relaxin action may be critical to the development of renal and systemic vasodilation in the first half of pregnancy, but as the placenta grows, placental vasodilators are the essential players in order to maintain the vasodilatory state thereafter. Kristiansson et al. showed low first trimester serum relaxin concentration were associated with increased blood pressure in the third trimester of pregnancy [33], but relaxin has not been investigated in respect to risk prediction.

To our knowledge, our study is the first study which investigated relaxin in a pathological pregnancy condition in rodents. Relaxin had no influence on blood pressure and proteinuria. A noteworthy limitation to our study is that we measured mean arterial blood pressure and not renal function as the measured experimental endpoint. We administered relaxin before the syndrome developed, and we have direct evidence that the peptide was increased substantially. The threshold dose of recombinant human relaxin in non pregnant rats to observe amelioration of kidney damage was 0.15 μg/h when given by subcutaneous osmotic minipump [34]. The infusion rate of our relaxin treatment had a 2 μg/h infusion rate, which should be in an efficient range. Nerveless we only had an elevation of around 5% of the endogenous relaxin amount. With this approach we observed a signifcant effect on heart rate, indicating that the approach needs to cardiovascular effects. The physiological increase in relaxin during pregnancy might be one reason why the intervention was not successful. To rule the effect of physiological relaxin elimination of relaxin from the circulation of pregnant rats, i.e., ovariectomy and subsequent maintenance of pregnancy with exogenous estradiol and progesterone before the start of the intervention would be important [35].

Furthermore we cannot exclude that an earlier start of relaxin therapy has a positive effect on endorgan damage. From previous studies we know that endovascular and interstitial trophoblast invasion, as well as trophoblast induced vascular remodeling of the spiral arteries are pathological in our model, which is one mechanism for the severe IUGR observed in our model [14, 15]. The spiral arteries that supply blood to the placental bed undergo significant morphological changes in normal pregnancy, including an increase in diameter and a concomitant decrease in smooth muscle cells resulted in thin-walled arteries [36]. The effects of relaxin on trophoblast function and vascular remodeling is sparse [37, 38], however they implicate that increasing the local and circulating relaxin concentrations will ameliorate the placental pathology. Relaxin did not improve the induced vascular remodeling of the pathologic spiral arteries in our model. The pathological placentation starts around day 4–5, whereas we start relaxin treatment at day 9. Relaxin is produced already very early during pregnancy by the corpus luteum and acts in an autocrine/paracrine fashion to influence granulosa/cumulus cell function [39]. Whether relaxin has direct effects on the embryo and/or trophoblast during implantation is not known, though certainly relaxin modulates the receptive endometrium. In a non-human primate model of early pregnancy relaxin stimulated uteroplacental vascularization by increasing number of arterioles in the endometrium [40]. The role of relaxin in physiological pregnancy in humans and rodents is not understood and species-dependent differences in relaxin biology might also be responsible for the study.

It appears counterintuitive, that the majority of the maternal circulation adaptation is complete at the end of the first trimester, when fetal and placental diameter is still small [41]. The cardiovascular changes are apparently preparatory anticipating the rapid growth phase of the fetus and placenta in the second and third half of gestation, when oxygen and nutrient demands rise enormously. Thus an earlier treatment with relaxin might be beneficial.

We have shown in earlier studies that anti-inflammatory therapies ameliorate renal and cardiac endorgan damage in the offspring of the pregnant rats. These rats harbour both human transgenes and develop massive hypertensive induced endorgan damage within 7–8 weeks, leading to a 50% mortality. In this model we also tested two different dosages of relaxin [42]. Both dosages tested did not ameliorate the renal and cardiovascular endorgan damage.

Our disappointment in the results has less to do with the science of relaxin but more is in terms of offering a therapeutic option for our patients. We believe that our data should not discourage investigators from performing such a trial. First, there are hardly such trials. The “undoable” trial was Magpie, a study to finally document the utility of magnesium sulphate [43]. Magnesium sulphate is a straightforward therapy accepted for 50 years. Other studies in preeclamptic women are more controversial and difficult. For instance, the removal of sFlt-1 (and possibly also antibodies directed against the Ang II receptor) showed preliminary encouraging results but await further investigation [44].

We could not substantiate the idea that in a rat model of preeclampsia relaxin would ameliorate the disease. An alternative approach to test the effect of relaxin would be to perform ovarectomy and sustain pregnancy by adding the necessary hormons to maintain pregnancy. With this approach relaxin differences between the groups will be expected to be much higher, leading to different results. We decided to perform the study as presented since the approach is closer to the human situation, where relaxin is considered to be applied. Thus, we believe our data give an additional impetus in a forward direction.
